# The Effect of Preexisting Immunity on Virus Detection and Immune Responses in a Phase II, Randomized Trial of a Russian-Backbone, Live, Attenuated Influenza Vaccine in Bangladeshi Children

**DOI:** 10.1093/cid/ciy1004

**Published:** 2018-11-27

**Authors:** Elizabeth B Brickley, Peter F Wright, Alexey Khalenkov, Kathleen M Neuzil, Justin R Ortiz, Larisa Rudenko, Min Z Levine, Jacqueline M Katz, W Abdullah Brooks

**Affiliations:** 1 Department of Infectious Disease Epidemiology, London School of Hygiene & Tropical Medicine, United Kingdom; 2 Department of Epidemiology, Geisel School of Medicine, Dartmouth College, Hanover; 3 Department of Pediatrics, Dartmouth-Hitchcock Medical Center, Lebanon, New Hampshire; 4 Center for Vaccine Development, University of Maryland School of Medicine, Baltimore; 5 Department of Virology, Institute of Experimental Medicine, Saint Petersburg, Russia; 6 Influenza Division, Centers for Disease Control and Prevention, Atlanta, Georgia; 7 Department of International Health, Johns Hopkins University, Baltimore, Maryland

**Keywords:** influenza vaccine, humoral immunity, mucosal immunity, immunogenicity

## Abstract

**Background:**

In a 2012 Phase II clinical trial, 300 Bangladeshi children aged 24 to 59 months with no prior influenza vaccine exposure were randomized to receive a single intranasally-administered dose of either trivalent, Russian-backbone, live, attenuated influenza vaccine (LAIV) or placebo. Protocol-defined analyses, presented in the companion manuscript, demonstrate decreased viral detection and immunogenicity for A/H1N1pdm09, relative to the A/H3N2 and B strains. This post hoc analysis of the trial data aims to investigate the LAIV strain differences by testing the hypothesis that preexisting humoral and mucosal immunity may influence viral recovery and immune responses after LAIV receipt.

**Methods:**

We used logistic regressions to evaluate the relations between markers of preexisting immunity (ie, hemagglutination inhibition [HAI], microneutralization, and immunoglobulin G and immunoglobulin A (both serum and mucosal antibodies) and LAIV viral recovery in the week post-vaccination. We then tested for potential effect modification by baseline HAI titers (ie, <10 versus ≥10) and week 1 viral recovery on the LAIV-induced serum and mucosal immune responses, measured between days 0 and 21 post-vaccination.

**Results:**

Higher levels of preexisting immunity to influenza A/H3N2 and B were strongly associated with strain-specific prevention of viral shedding upon LAIV receipt. While evidence of LAIV immunogenicity was observed for all 3 strains, the magnitudes of immune responses were most pronounced in children with no evidence of preexisting HAI and in those with detectable virus.

**Conclusions:**

The results provide evidence for a bidirectional association between viral replication and immunity, and underscore the importance of accounting for preexisting immunity when evaluating virologic and immunologic responses to LAIVs.

**Clinical Trials Registration:**

NCT01625689.

## Teaser

Even in previously vaccine-naive populations, preexisting immunity, such as that potentially arising from prior exposure to natural infection, may influence vaccine virus recovery and immune responses following receipt of a live, attenuated influenza vaccine.

**(See the Major Article by Lewis et al on pages 777–85, and the Editorial Commentary by Belshe on pages 795–6.)**

Owing to the ease of their intranasal administration and relatively low cost, live, attenuated influenza vaccines (LAIVs) have the potential to be valuable tools for seasonal and pandemic influenza prevention, particularly in low-resource settings [1]. Nevertheless, a key challenge for LAIVs is that they have highly-variable effectiveness [2], which may be modulated by construct-related differences (eg, similarity to circulating influenza viruses or inherent variation in the extent of attenuation of recombinant viruses) and host-specific factors (eg, age and health conditions). Whereas humoral, mucosal, and cell-mediated immunologic responses have been found to be associated with LAIV receipt in some cases [3–5], no specific combination of immune parameters has been consistently shown to be a true correlate of protection for LAIVs against influenza virus infection [6, 7]. Accumulating evidence indicates that an underlying driver of the heterogeneity in immunologic responses to influenza vaccines in children may, in fact, be their prior exposure to naturally-occurring infections or other influenza vaccines [8–10]. A better understanding of the effects of preexisting immunity on post-vaccination endpoints is needed to improve LAIV clinical evaluation and performance.

Using data from a 2012 Phase II, randomized, double-blind, parallel-group, placebo-controlled trial of a seasonal, trivalent, Russian-backbone LAIV (NCT01625689), this investigation aimed to shed new light on the interplay between preexisting immunity, vaccine virus detection, and LAIV-induced humoral and mucosal immune responses. For the trial, 300 healthy, influenza vaccine–naive children aged 24 to 59 months residing in Dhaka, Bangladesh, were randomized 1:1 to receive a single, intranasal dose of either a placebo control (Lot E9001PCB) or an A/Leningrad and B/USSR-backbone vaccine produced by the Serum Institute of India (Pune, India) [11] and containing the World Health Organization–recommended Northern Hemisphere influenza vaccine formulation for the 2011–2012 season (ie, A/California/7/2009 [H1N1] pdm09-like virus, A/Victoria/361/2011 [H3N2]-like virus, and B/Wisconsin/1/2010-like virus) [12]. A companion study by Lewis and colleagues reports the protocol-defined analyses, demonstrating that viral detection and immune responses in the LAIV recipients differed by vaccine strains, with no A/H1N1pdm09 viral recovery, as well as markedly decreased immunogenicity observed for A/H1N1pdm09, relative to the A/H3N2 and B strains [13]. Of note, previous work in Bangladesh has demonstrated a significant impact of influenza illness in young children [14], with a notable penetration of A/H1N1pdm09 virus, with its initial circulation in Dhaka [15]. The post hoc investigation reported here builds on the work by Lewis and colleagues by assessing whether children’s strain-specific preexisting immunities may influence the odds of vaccine virus detection in the week after vaccination and whether this preexisting immunity, as well as nasopharyngeal viral replication, may modify LAIV-induced serum and mucosal immune responses measured in the 21 days post-vaccination.

## METHODS

### Study Design and Laboratory Procedures

The study design and laboratory procedures are detailed in the companion manuscript [13]. Briefly, nasopharyngeal wash (NPW) specimens were collected from the children on trial days 0, 2, 4, 7, and 21 and stored frozen at −80°C; serum samples were collected on days 0 and 21 and stored frozen at −20°C. The origin of identified strains (ie, vaccine or wild-type) were confirmed in NPW specimens by reverse-transcription polymerase chain reaction [16]. Serum-neutralizing antibodies against A/H1N1pdm09, A/H3N2, and B influenza were measured using hemagglutination inhibition (HAI) and microneutralization (MN) assays; titers of strain-specific serum immunoglobulin (Ig) G and IgA were quantified using enzyme-linked immunosorbent assays [17, 18]. Titers of strain-specific mucosal IgA were quantified using kinetic, enzyme-linked immunosorbent assays and were normalized relative to the total specimen IgA [3, 4]. Out of the total of 300 participants, the 290 participants who had evaluable (ie, sample received and considered valid) reverse-transcription polymerase chain reaction typing of NPW specimens for each of the 3 time points in the week following vaccination (ie, days 2, 4, and 7) were selected for inclusion in the analytical cohort for the current investigation [16].

### Ethics and Role of the Funding Source

The study was approved by the Committee for the Protection of Human Subjects at Dartmouth College (Hanover, NH), the Western Institutional Review Board (Olympia, WA), and the local ethical review board of the International Centre for Diarrheal Disease Research, Bangladesh (Dhaka, Bangladesh), and was conducted in accordance with the Declaration of Helsinki, the International Conference on Harmonisation guideline for Good Clinical Practice, and the codes and regulation of the United States and Bangladesh regarding research on human subjects. The initial consent included provisions for the use of samples in future influenza-related studies.

### Statistical Approaches

Pairwise correlations between baseline (ie, trial day 0, prior to vaccination) levels of strain-specific serum and mucosal immune markers were estimated in the full analytical cohort with Spearman’s rank correlation coefficients and were visualized in matrices using the corrplot R package, version 0.77 [19]. LAIV recipients were categorized as either shedding-positive or shedding-negative, based on their A/H3N2 and B viral detection status (ie, by whether any or no virus was detected from NPW specimens on days 2, 4, and 7 after LAIV receipt); of note, no A/H1N1pdm09 shedding was detected in the NPW specimens on days 2, 4, or 7 after LAIV receipt. The associations between baseline immunity to A/H3N2 and B influenza (ie, indicated by a tertile of the strain-specific immune marker on trial day 0) and the odds of being shedding-negative were investigated using logistic regressions. In order to allow the effect sizes to be compared informatively across any pair of tertiles and without depending on the precision within an arbitrarily-selected baseline group, 95% confidence intervals (CIs) were estimated from floated variances [20]. The results were visualized by plotting the odds ratios and 95% CIs on the y-axis, versus the geometric mean for each tertile of immune marker on the x-axis. Cross-sectional differences in the log_2_ A/H3N2- and B-specific immune marker levels on trial days 0 and 21 were compared between the shedding-negative and shedding-positive groups using *t*-tests. For all 3 strains, immune responses following LAIV receipt were visualized in the placebo, LAIV shedding-negative, and LAIV shedding-positive groups using scatterplots; *P* values were calculated separately within each treatment/shedding group using paired *t*-tests, comparing the log_2_ titers on trial days 0 and 21. Density plots were used to visualize the distributions of differences in the log_2_ serum and mucosal antibody titers between trial days 0 and 21 by immunoassay, influenza strain, and treatment/shedding status, as well as treatment/baseline HAI status (ie, by whether pre-vaccination HAI titers were <10 or ≥10). Effect modification in the immune responses by (1) baseline HAI status and (2) week 1 viral recovery were evaluated using multiplicative interaction terms in multilevel, mixed-effects, linear regressions that allowed for participant-specific random effects; *P* values indicating the statistical significance of the interaction term are from likelihood ratio tests. To mitigate the potential for false-positive results, a Bonferroni-corrected *P* value of .0007 (ie, .05 ÷ 70) was considered to be the threshold for significance. All *P* values are from 2-sided statistical tests, and all analyses were performed using Stata, version 13.0 (StataCorp LP, College Station, TX) and R, version 3.2.5.

## RESULTS

The relationships between preexisting immunity, vaccine virus detection, and LAIV-induced humoral and mucosal immune responses were examined in an analytical cohort comprising 145 LAIV recipients and 145 placebo controls, which represented 97% of the participants in the primary study [12]. At baseline, strong pairwise correlations were observed for the levels of strain-specific serum immune markers (*P* < .0001 for all; Supplementary Figure 1). In each of the 3 strains, the most highly-correlated markers were microneutralization and serum IgG (Spearman’s rho: A/H1N1pdm09, 0.82; A/H3N2, 0.78; B, 0.77). As reported in the companion, protocol-defined analyses, viral shedding patterns and immune responses in LAIV recipients differed by vaccine strains. Whereas the A/H1N1pdm09 virus was not detected over follow-up, the A/H3N2 and B vaccine viruses were detected in NPW specimens on 1 or more days from 46% (n = 67/145) and 59% (n = 86/145) of vaccinees, respectively. Of note, wild-type B viruses were also detected by Sanger sequencing in 3 placebo recipients, demonstrating the presence of intercurrent influenza B in circulation in Dhaka at the time of the trial.

### Baseline Immune Marker Levels and the Odds of Not Shedding Virus Upon Vaccine Receipt

**Figure 1.**
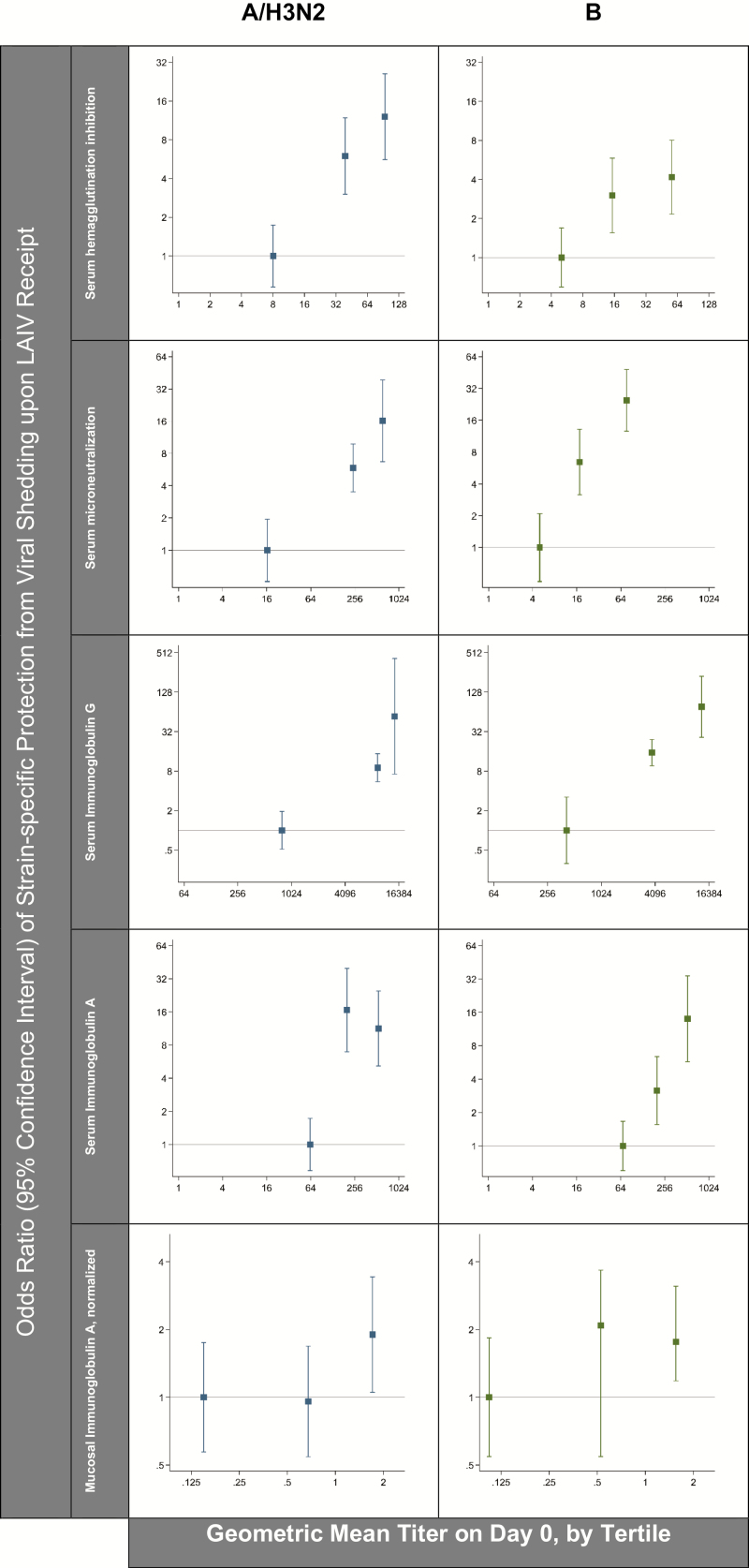
The association between preexisting immunity (ie, indicated by a tertile of the strain-specific immune marker on trial day 0) and subsequent protection from strain-specific viral shedding upon LAIV receipt (ie, indicated by no viral recovery from NPW specimens on trial days 2, 4, and 7; n = 145). Odds ratios for being shedding-negative (y-axis) are plotted versus the mean for each tertile of the immune marker at baseline (x-axis). The horizontal reference line indicates an odds ratio of 1. Abbreviations: LAIV, live, attenuated influenza vaccine; NPW, nasopharyngeal wash.

In comparing the vaccine recipients in whom a virus was detected versus those without virus detection for A/H3N2 and B (Table 1), the baseline titers of all of the measured serum immune markers were statistically significantly lower in the children from whom virus was detected after LAIV receipt. To examine the shape of the associations more directly, vaccine recipients were grouped into tertiles based on their preexisting antibody titers (Supplementary Table 1), and the odds of being shedding-negative were compared between the groups (Figure 1). Across the panel of serum immune markers, the results consistently indicate the odds of not shedding a virus upon vaccine receipt increased progressively with each incremental change in preexisting immune titers. For both A/H3N2 and B mucosal IgA, children in the highest third had approximately 2-fold increased odds of being shedding-negative upon LAIV receipt, relative to children in the lowest third.

**Table 1.** Cross-sectional Comparison of Strain-specific Serum and Mucosal Antibody Titers

		A/H1N1pdm09LAIV Shedding- negative Group (n = 145)	A/H3N2LAIV Shedding- negative Group (n = 78)	A/H3N2LAIV Shedding-positive Group (n = 67)		BLAIV Shedding-negative Group (n = 59)	BLAIV Shedding-positive Group (n = 86)	
	Day	Geometric Mean Titer (95% CI)	Geometric Mean Titer (95% CI)		*P* Value,Between Shedding Groups	Geometric Mean Titer (95% CI)		*P* Value,Between Shedding Groups
Serum HAI	0	18 (15–22)	46 (37–56)	13 (9.8–16)	<.0001	18 (13–24)	9.4 (7.7–11.5)	.0003
	21	25 (21–31)	72 (58–89)	71 (52–97)	.93	49 (35–69)	25 (19–34)	.003
Serum microneutralization	0	49 (38–64)	270 (210–340)	48 (31–73)	<.0001	49 (29–53)	8.7 (7.1–10.6)	<.0001
	21	65 (50–86)	370 (300–460)	390 (270–570)	.79	86 (63–120)	34 (25–48)	.0002
Serum immunoglobulin G	0	4400 (3400–5600)	8100 (6600–9800)	1700 (1200–2500)	<.0001	5800 (4700–8800)	1100 (860–1500)	<.0001
	21	6200 (4900–7800)	10 000 (8600–11 000)	12 000 (9700–15 000)	.13	10 000 (8300–12 000)	8600 (7100–10 000)	.26
Serum immunoglobulin A	0	190 (150–220)	240 (200–290)	85 (71–100)	<.0001	230 (180–430)	92 (80–110)	<.0001
	21	270 (220–330)	390 (320–480)	300 (220–390)	.10	440 (340–570)	350 (280–450)	.23
Mucosal immunoglobulin A, normalized	0	0.5 (0.4–0.6)	0.6 (0.5–0.8)	0.5 (0.3–0.6)	.09	0.5 (0.3–0.6)	0.4 (0.3–0.6)	.63
	21	0.5 (0.4–0.6)	0.8 (0.6–1.1)	0.8 (0.6–1.1)	.86	0.7 (0.5–1.0)	0.6 (0.5–0.9)	.72

Comparisons are between shedding-negative and shedding-positive groups, stratified by trial day in LAIV recipients (n = 145). *P* values are from *t*-tests of the log_2_ titers.

Abbreviations: CI, confidence interval; HAI, hemagglutination inhibition; LAIV, live, attenuated influenza vaccine.

### Immune Responses to Vaccination by Treatment and Viral Shedding Status

**Figure 2.**
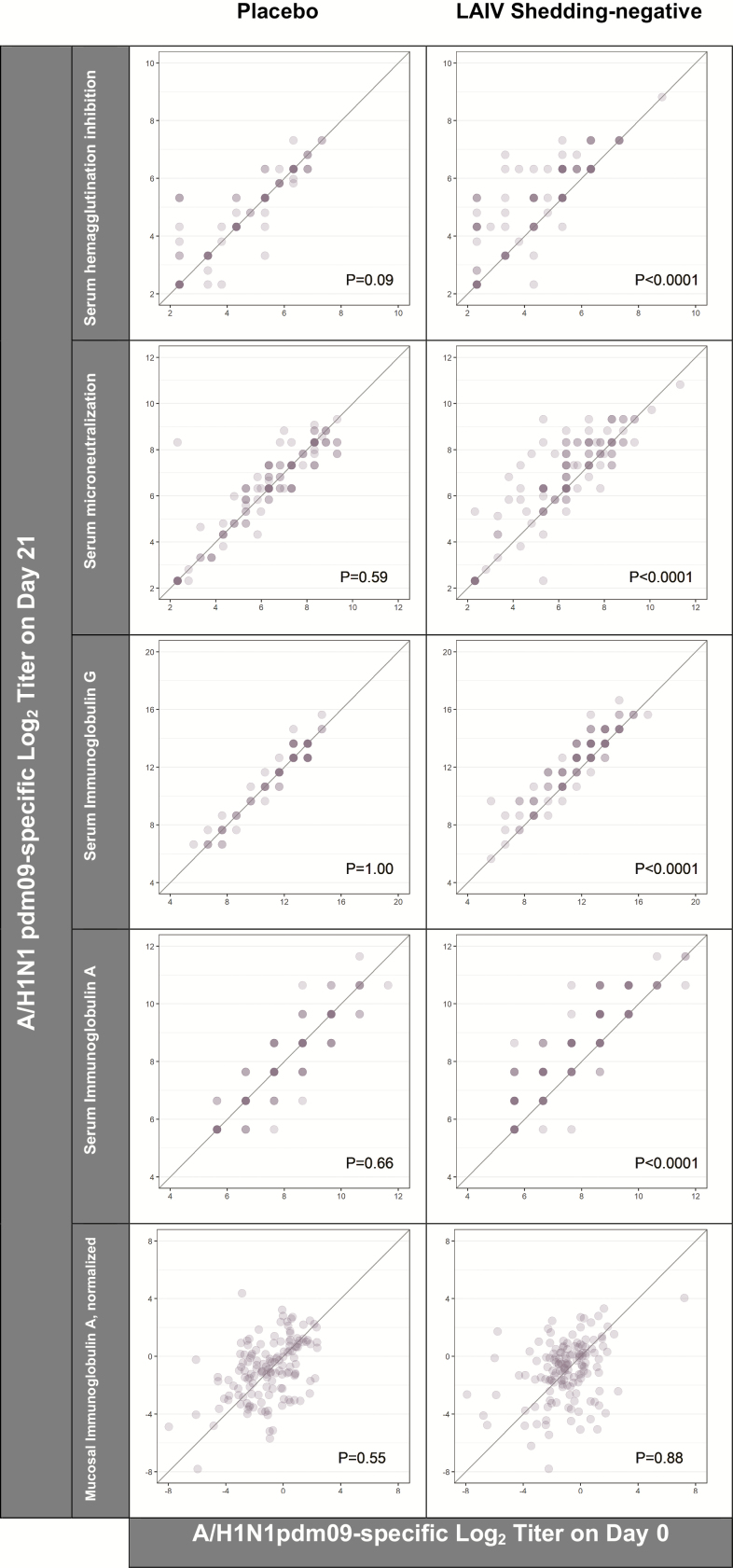
A/H1N1pdm09-specific immune responses to placebo (n = 145) and LAIV receipt (n = 145). Of LAIV recipients, 100% were categorized as A/H1N1pdm09 shedding-negative (ie, indicated by no viral recovery on trial days 2, 4, and 7). The intensity of the color indicates the number of individuals at a given coordinate. *P* values are from paired *t*-tests comparing the log_2_ titers on trial days 0 and 21. The diagonal reference line indicates equivalence between the time points. Abbreviation: LAIV, live, attenuated influenza vaccine.

**Figure 3.**
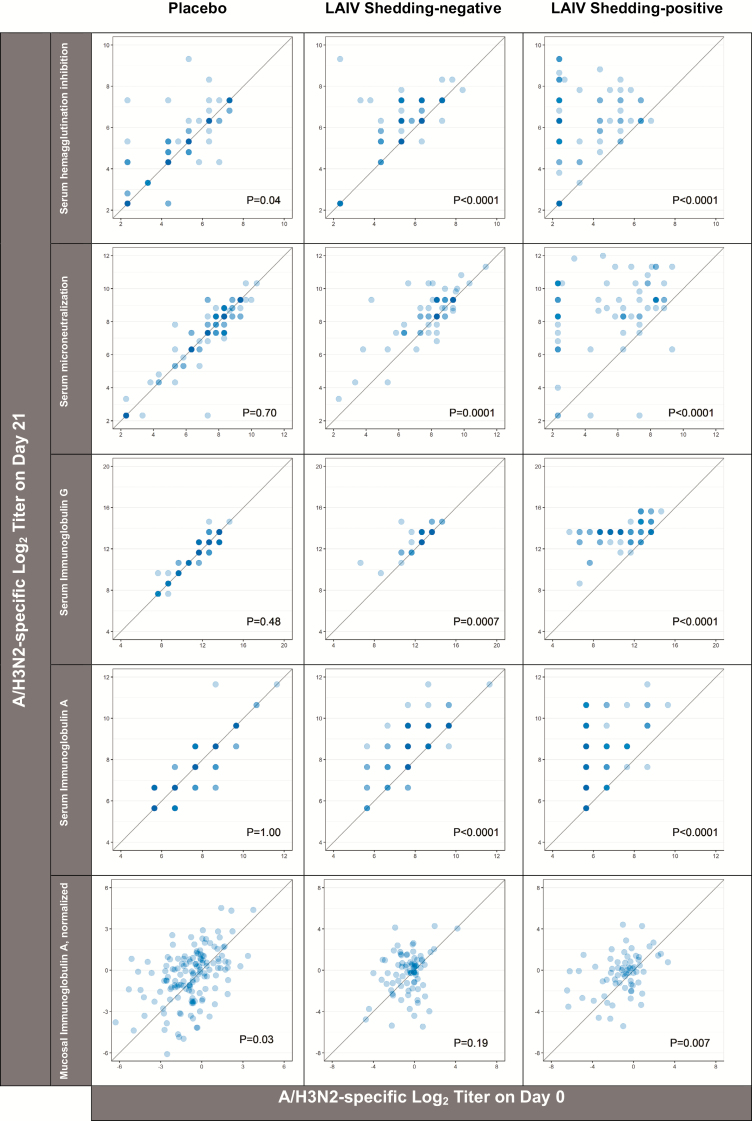
A/H3N2-specific immune responses to placebo (n = 145) and LAIV receipt (n = 145). Of LAIV recipients, 46% were categorized as A/H3N2 shedding-negative (ie, indicated by no viral recovery on trial days 2, 4, and 7). The intensity of the color indicates the number of individuals at a given coordinate. *P* values are from paired *t*-tests comparing the log_2_ titers on trial days 0 and 21. The diagonal reference line indicates equivalence between the time points. Abbreviation: LAIV, live, attenuated influenza vaccine.

**Figure 4.**
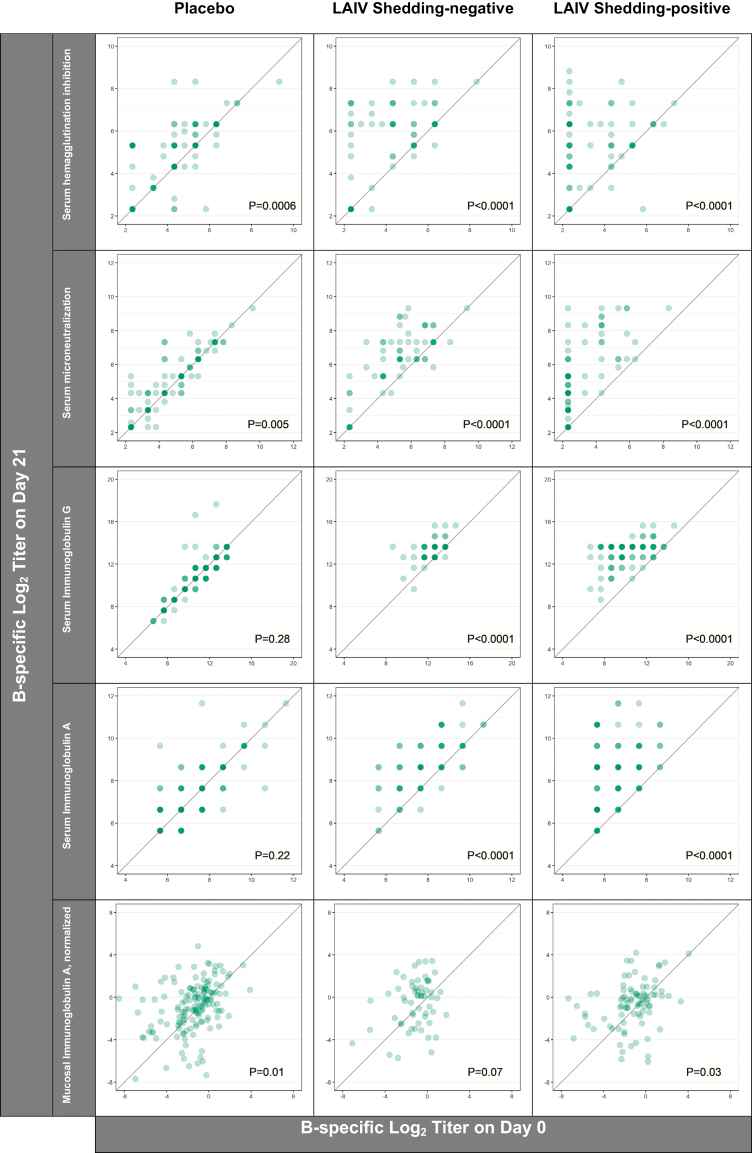
B-specific immune responses to placebo (n = 145) and LAIV receipt (n = 145). Of LAIV recipients, 59% were categorized as A/H3N2 shedding-negative (ie, indicated by no viral recovery on trial days 2, 4, and 7). The intensity of the color indicates the number of individuals at a given coordinate. *P* values are from paired *t*-tests comparing the log_2_ titers on trial days 0 and 21. The diagonal reference line indicates equivalence between the time points. Abbreviation: LAIV, live, attenuated influenza vaccine.

The 145 eligible placebo recipients had no detectable virus and no significant rises in humoral and mucosal immune markers between trial days 0 and 21 for either A/H1N1pdm09 (Figure 2) or A/H3N2 (Figure 3). In contrast, placebo recipients exhibited statistically-significant rises in B-specific serum HAI, likely reflecting environmental exposure to wild-type B viruses over follow-up (Figure 4).

Varying degrees of LAIV immunogenicity were observed for all 3 strains. Despite the absence of A/H1N1 virus detection after vaccination, LAIV recipients did have marginally higher levels of serum immune markers on day 21 than on day 0 (ie, when measured as a continuous variable rather than by a 4-fold rise, as reported in [13]; Figure 2). Serum and mucosal titers for A/H3N2 rose between days 0 and 21 in both the shedding-negative and -positive groups (Figure 3). Similarly, with the exception of mucosal IgA levels, B-specific immune markers rose statistically significantly after LAIV receipt (Figure 4). Relative to the serum markers, a greater degree of variance was observed in the measures of mucosal IgA for each of the 3 LAIV viruses.

**Figure 5.**
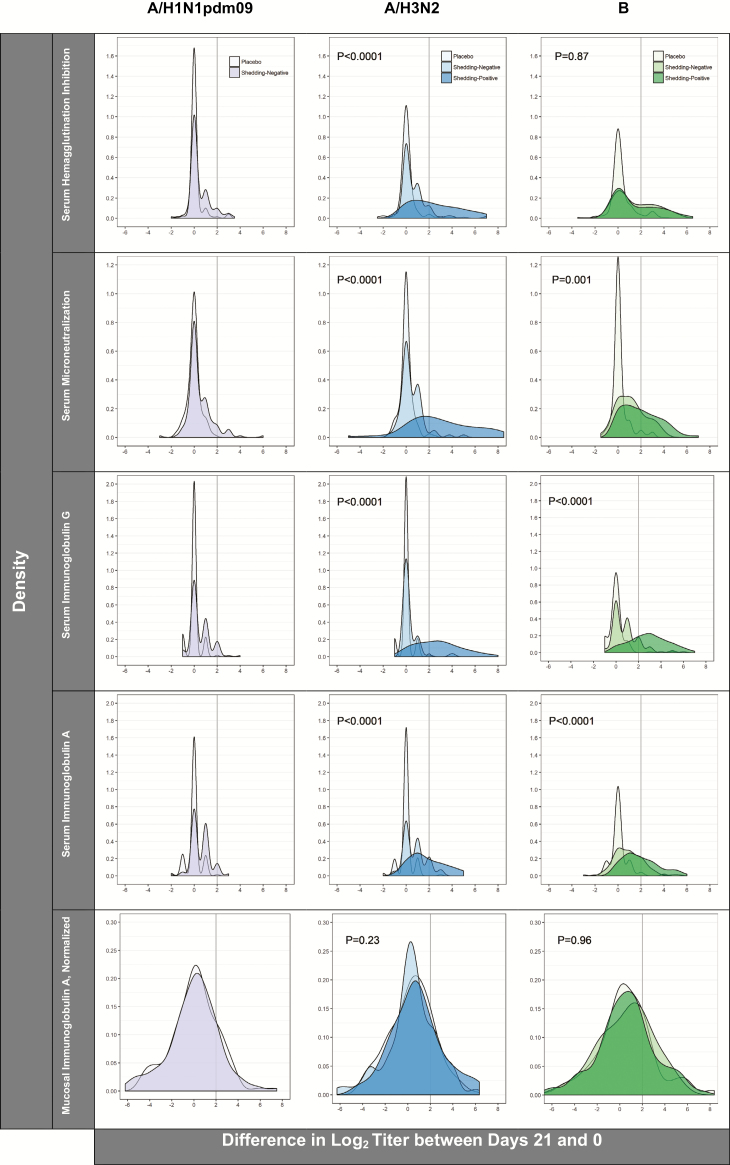
Density plots illustrating the distributions of differences in the log_2_ serum and mucosal antibody titers between trial days 0 and 21 by immunoassay, influenza strain, and treatment/shedding status (N = 290). *P* values are from likelihood ratio tests and indicate effect modification of the immune responses by shedding status (ie, shedding-negative versus shedding-positive). The vertical reference lines indicate 4-fold rises (ie, a log_2_ difference ≥2) between the time points.

Subsidiary analyses demonstrated that there was significant effect modification in the serum immune responses between trial days 0 and 21 in children with preexisting HAI titers below 10 and in those with detectable, strain-specific viral shedding. As illustrated in Supplementary Figure 2, post-vaccination rises in A/H3N2-specific HAI, MN, and IgG titers and in B-specific IgG titers were more pronounced in children with baseline HAI titers below 10, relative to their peers with baseline HAI titers above 10. Similarly, post-vaccination rises for all A/H3N2-specific serum markers and also for B-specific serum IgG and IgA differed by shedding status, such that a greater proportion of children in the shedding-positive group experienced 4-fold rises than their peers in the shedding-negative category (Figure 5). Cross-sectional analyses from trial day 21 indicate that, although the magnitudes of the responses between days 0 and 21 were influenced by shedding category, the A/H3N2 titers achieved following vaccination with the LAIV did not differ substantively between the shedding and non-shedding groups (Table 1). For the influenza B component, the day 21 geometric mean titers of the serum and mucosal immune markers remained higher in the non-shedders, potentially reflecting some degree of preexisting immunity from prior, wild-type infection in the shedding-negative group (Table 1).

## DISCUSSION

The current investigation is an extension of a collaborative international effort to examine the safety [13], virus shedding, and immune responses [13] of Bangladeshi children to a Russian-backbone, live, attenuated, intranasally-delivered vaccine in a Phase II trial. The results presented here provide evidence that vaccinees with lower baseline immunity to influenza A/H3N2 and B (ie, as indicated by lower pre-vaccination antibody titers) have significantly higher odds of shedding a strain-specific virus in the week after vaccination. Further, while the magnitudes of the A/H3N2- and B-specific immune responses to vaccination were significantly more pronounced in participants with detectable vaccine virus shedding (Figure 5), LAIV-induced systemic immune markers to A/H3N2 and B influenza also rose statistically significantly in participants with no detectable vaccine virus shedding. Ultimately, the geometric mean titers of serum antibody attained by day 21 in the shedding-positive groups were relatively similar to, in the case of A/H3N2, and somewhat lower than, in the case of B, the respective shedding-negative groups, which generally started with higher preexisting antibody titers. Overall, the findings from the A/H3N2 and B influenza strains suggest viral shedding upon LAIV receipt may have greater utility as an indicator of an individual’s immunologic experience than as a generalizable correlate of LAIV immunogenicity.

Given these observations, the lack of A/H1N1pdm09 virus detection, despite modest serum antibody responses following LAIV receipt, is particularly noteworthy. As high rates of A/H1N1 infections were reported in Bangladesh during the 2009 pandemic [15], preexisting immunity generated from prior natural infection may have influenced the trial’s A/H1N1pdm09 results to some degree. Several pieces of evidence support this hypothesis. First, like those for A/H3N2, the geometric mean titers for A/H1N1pdm09 were consistently higher than the titers measured for B at baseline (Table 1). Second, comparing the density plots of the differences in log_2_ titers between days 21 and 0 across strains (Figure 5), strikingly similar distributions are present in the shedding-negative groups for A/H1N1pdm09 and A/H3N2. Third, the results from a subsequent Phase III Bangladeshi trial of the same A/H1N1pdm09 Russian-backbone component showed a 50% vaccine efficacy for A/H1N1pdm09, despite the limited shedding and immune responses measured in this Phase II trial [21]. Taken together, these findings reinforce the idea that, in the context of recently- or currently-circulating viruses, some clinically-relevant immunity may be induced by LAIVs, even in the absence of demonstrable virus replication [9, 22]. Such immune response without detectable virus replication is surprising, but has been previously observed with respiratory syncytial virus in an animal model [21] and in a human challenge study [23]. There are 3 possible explanations: the first is that replication occurred at a low level or different anatomic site from that sampled; the second is that the antigenic load presented by the initial dose of vaccine stimulated immunity; or the third is that we do not yet understand the true correlate(s) of immunity to influenza.

It is also plausible that additional vaccine-related features, such as strain-specific replicative fitness, genetic stability, and/or virus competition for cell receptors, may have contributed to the lack of detectable A/H1N1pdm09 replication. Importantly, good growth of A/H1N1 vaccine virus was originally observed in the independently-derived Ann Arbor–backbone LAIV [24, 25], which was accompanied by proof of efficacy in United States [26]. However, the Ann Arbor A/H1N1 more recently has exhibited a lack of effectiveness [27], leading to discontinuation of the Centers for Disease Control and Prevention recommendation of its use for the 2016–2017 and 2017–2018 influenza seasons in the United States [28]. Although only 1 direct comparison of safety, infectivity, and immunogenicity of the A/Ann Arbor and A/Leningrad vaccines has been carried out to date [29], it would appear from this experience that the 2 master strains have a similar level of attenuation.

The unique analytical approach of this study introduced both strengths and limitations. As LAIVs do not have an established correlate of protection, immunogenicity to influenza has conventionally been evaluated in terms of 4-fold rises in serum HAI and/or the achievement of a protective titer, typically of at least 40. Earlier research has found, against the backdrop of both vaccine- and naturally-induced immunity in children in the United States, that serum HAI is a poor predictor of susceptibility to A/Ann Arbor vaccine infection [6] and that the serum antibody generated in response to an inactivated vaccine appears to be minimally protective on a live vaccine challenge [3]. Considering this, we took a different approach in this analysis, evaluating humoral and mucosal immune responses as a function of the vaccine or placebo group assignment, the presence or absence of preexisting neutralizing antibodies, and the presence or absence of vaccine virus recovery. While the methods used here provide new insight into the findings from Lewis and colleagues [13] by first estimating the associations between preexisting B cell–mediated humoral and mucosal immunity and the odds of viral detection after LAIV administration and then comparing the responses of those same immune parameters to vaccination, given strain-specific baseline HAI titers and viral detection, further investigations in other study settings are needed to replicate these findings. In addition to being conducted in a setting, Bangladesh, where enteric pathogens and microflora differ from developed country settings [30], the influenza immunity in this population arose exclusively from prior exposure to wild-type virus. It differs from the experience in the United States, where correlates of immunity are difficult to measure after exposure to influenza antigens from both natural infection and vaccination [6]. Further, whereas mucosal IgA levels measured in stool samples have been previously shown to provide a robust indication of vaccine-induced protection in the context of polio (eg, see [31, 32]), the mucosal IgA levels measured in NPW specimens in this study exhibit a high degree of variance that limits their utility; further research is needed to optimize NPW sampling methods and refine the ascertainment of influenza-specific mucosal IgA.

In conclusion, the study shows good infectivity and immunogenicity of the A/H3N2 and B Russian-backbone strains in Bangladesh, particularly in those whose low level of immunity suggested that the LAIV was their first influenza exposure. As such, they add to the safety and immunogenicity data supporting the use of LAIVs on a global basis. The results of this study also illustrate that, when viewed alone, neither virus recovery nor immunologic response are fully predictive of a protective response to LAIVs. Even in previously vaccine-naive populations, accounting for preexisting immunity, such as that potentially arising from prior exposure to natural infection, is essential for interpreting a LAIV’s virologic and immunologic take.

## Supplementary Data

Supplementary materials are available at *Clinical Infectious Diseases* online. Consisting of data provided by the authors to benefit the reader, the posted materials are not copyedited and are the sole responsibility of the authors, so questions or comments should be addressed to the corresponding author.

ciy1004_suppl_Supplementary_Material

## Notes

***Acknowledgments.*** The authors thank F. Liani Gross, Zhunan Li, Stacie Jefferson, Jessica Trost, and David Wang from the Influenza Division of the Centers for Disease Control and Prevention for their assistance in conducting serum immunogenicity assays (hemagglutination inhibition, microneutralization, immunoglobin G, and immunoglobin A enzyme-linked immunosorbent assays).

***Financial support.*** This work was supported by funding from the Bill & Melinda Gates Foundation, which provides financial support to PATH’s Influenza Vaccine Project (grant number OPP48805).

***Potential conflicts of interest.*** K. M. N. received funding from the Bill and Melinda Gates Foundation for the Typhoid Vaccine Acceleration Consortium project. All other authors report no potential conflicts. All authors have submitted the ICMJE Form for Disclosure of Potential Conflicts of Interest. Conflicts that the editors consider relevant to the content of the manuscript have been disclosed.
