# Supplementary material for: The Effect of Preexisting Immunity on Virus Detection and Immune Responses in a Phase II, Randomized Trial of a Russian-Backbone, Live, Attenuated Influenza Vaccine in Bangladeshi Children
Source: Clin Infect Dis. 2018 Nov 27;69(5):786–94. doi: 10.1093/cid/ciy1004 (PMC6695513; doi:10.1093/cid/ciy1004)
Supplement: ciy1004_suppl_Supplementary_Material [file ciy1004_suppl_supplementary_material.docx]

**Supplementary Figure 1. Pairwise correlations between serum and mucosal immune markers specific to (A) A/H1N1, (B) A/H3N2, and (C) B influenza strains measured on trial day 0 (N=290).** Correlations were estimated by Spearman’s rank. The narrowness of the ellipse and intensity of the color indicate the strength of a given correlation coefficient. Diagonal lines indicate perfect correlations. The corresponding numerical values are defined by the vertical bar on the right. Abbreviations: HAI, hemagglutination inhibition; MN, microneutralization; sIg, serum immunoglobulin; mIg, mucosal immunoglobulin.

A
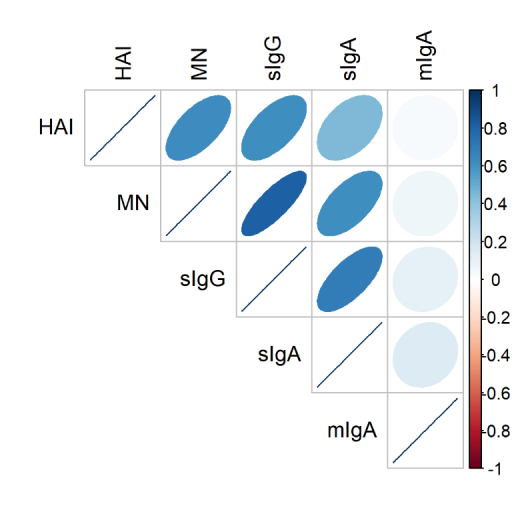
 B
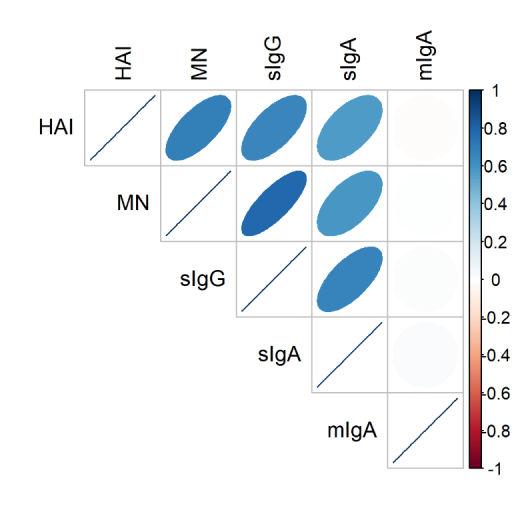
 C
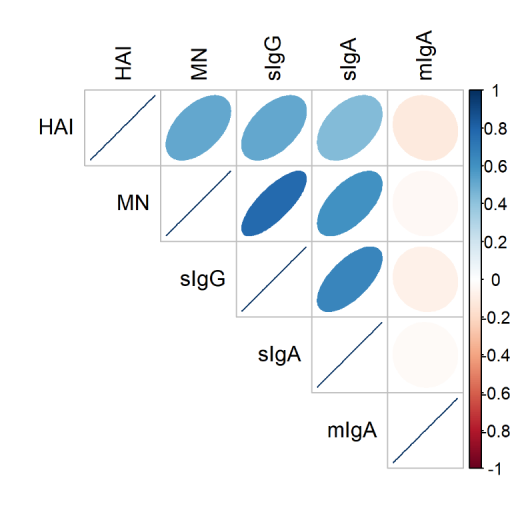


|  |  | **A/H1N1pdm09** | **A/H3N2** | **B** |
| --- | --- | --- | --- | --- |
| **Density** | **Serum Hemagglutination Inhibition** | 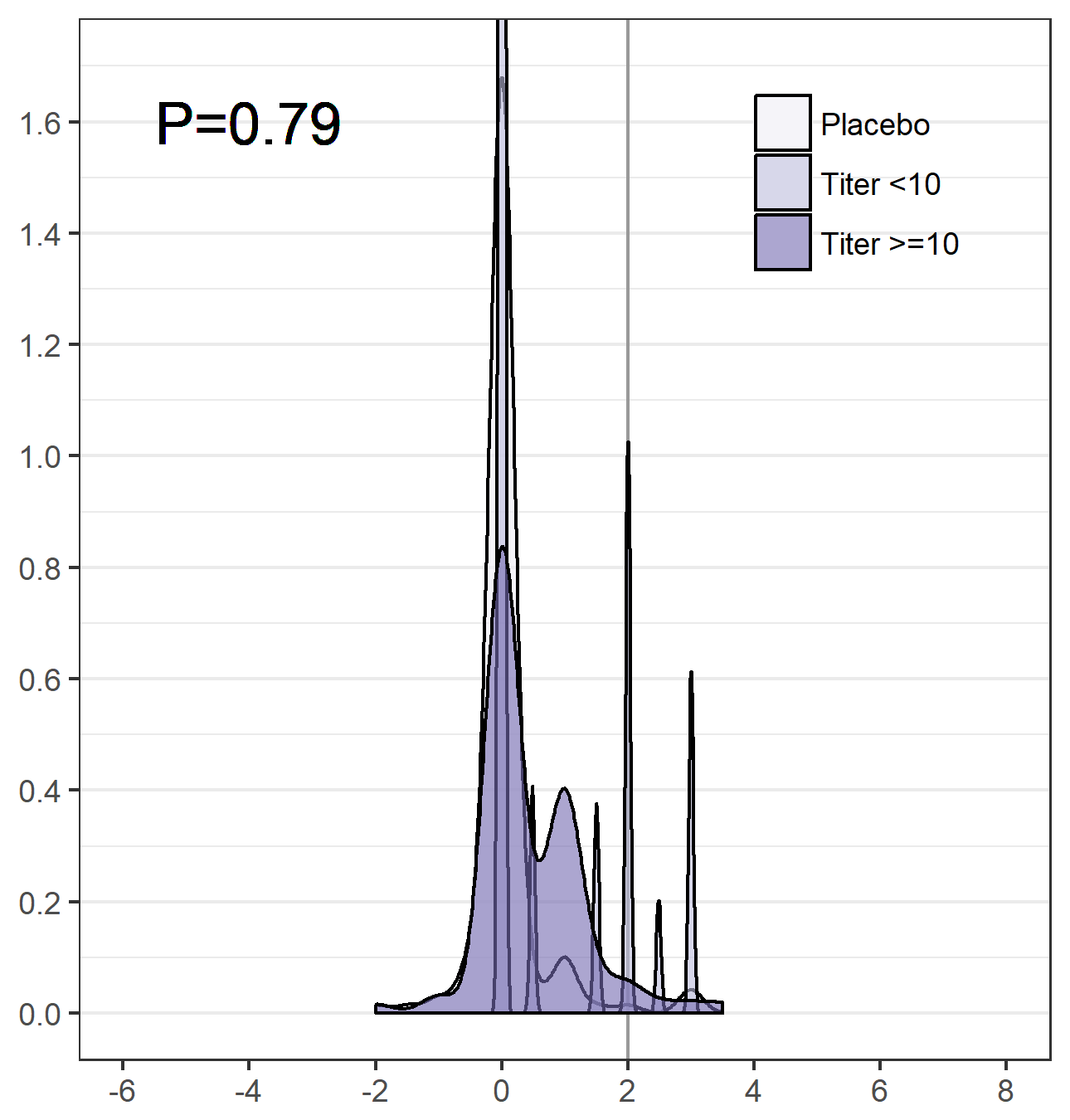 | 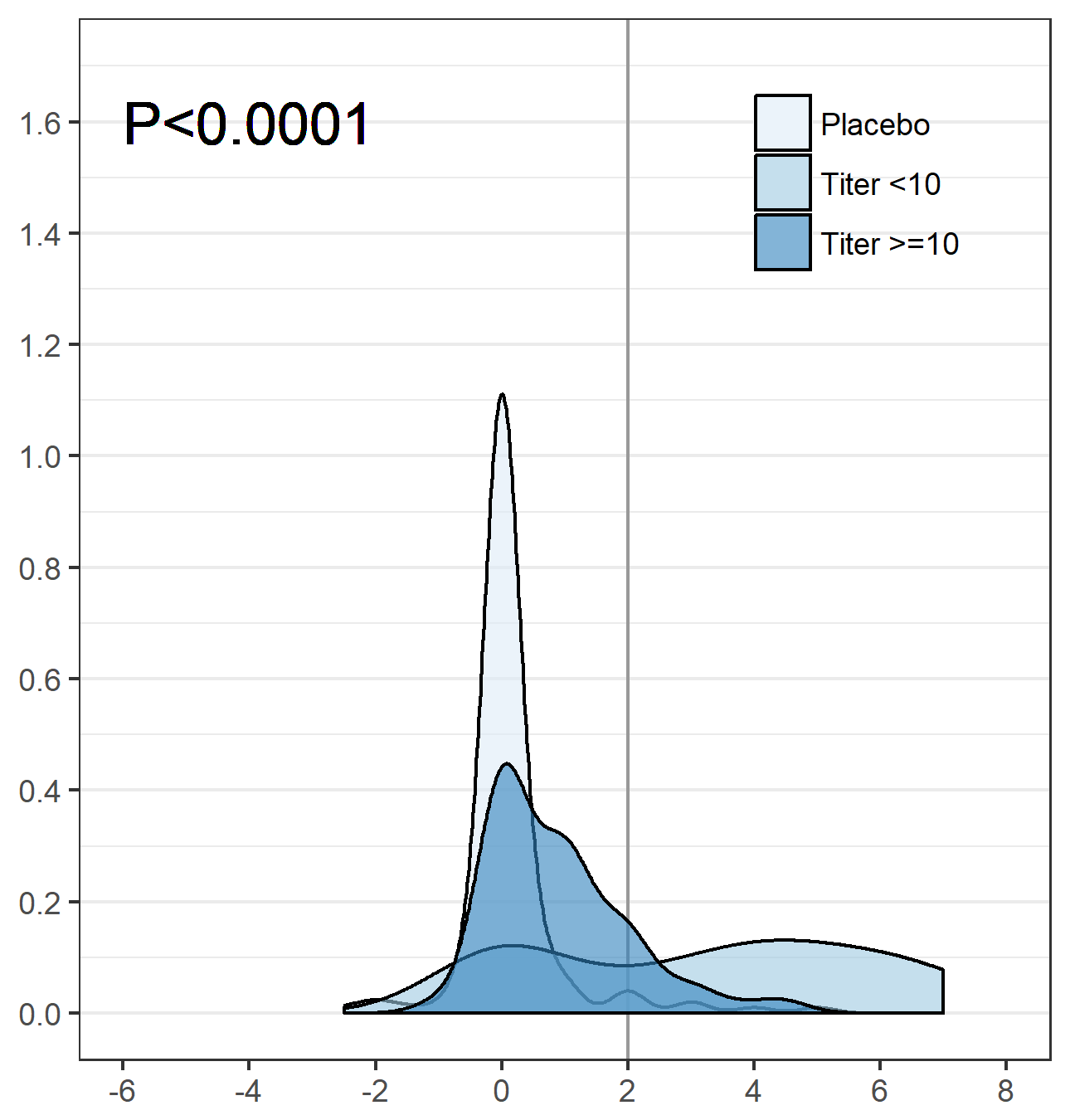 | 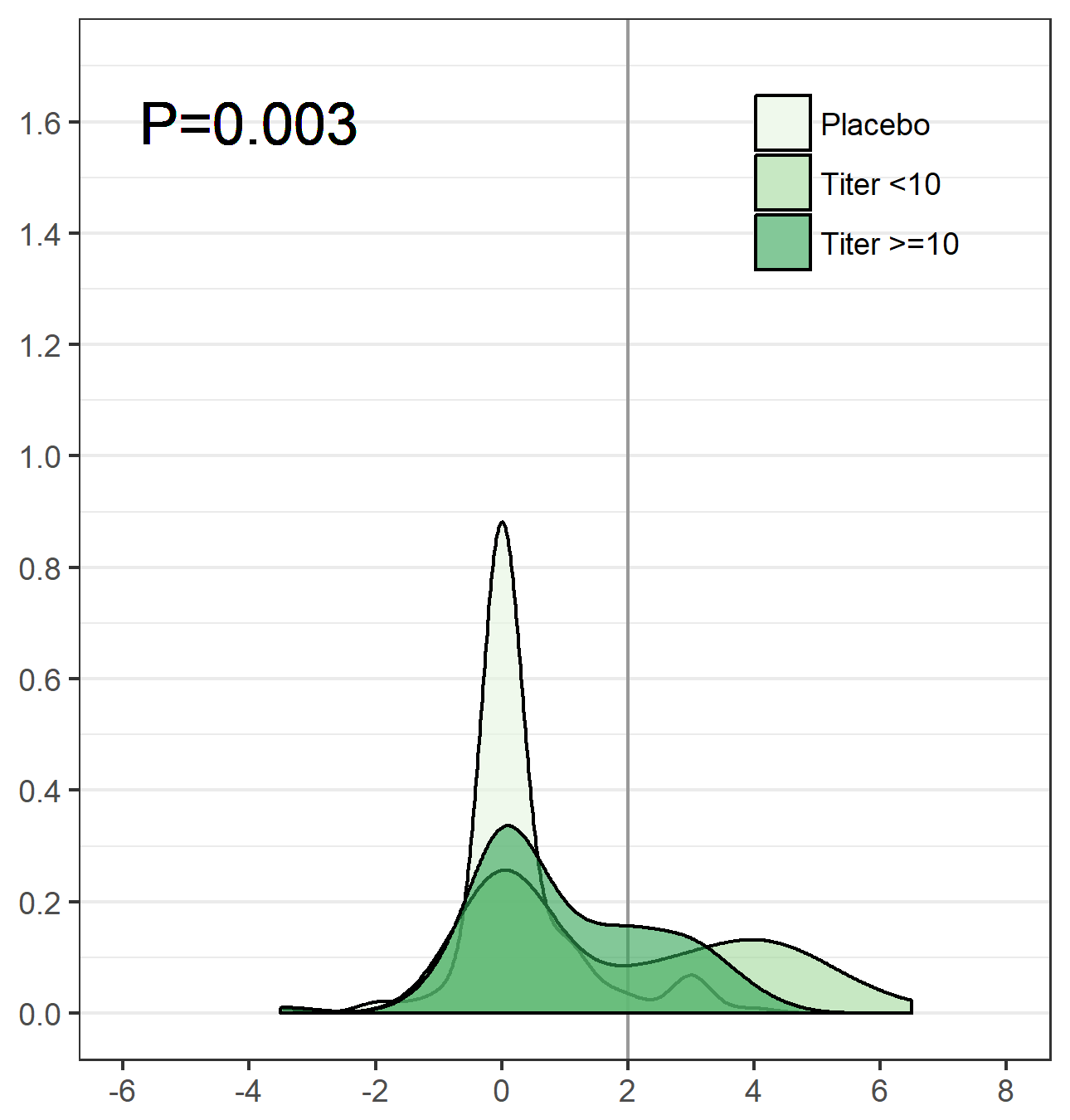 |
|  | **Serum Microneutralization** | 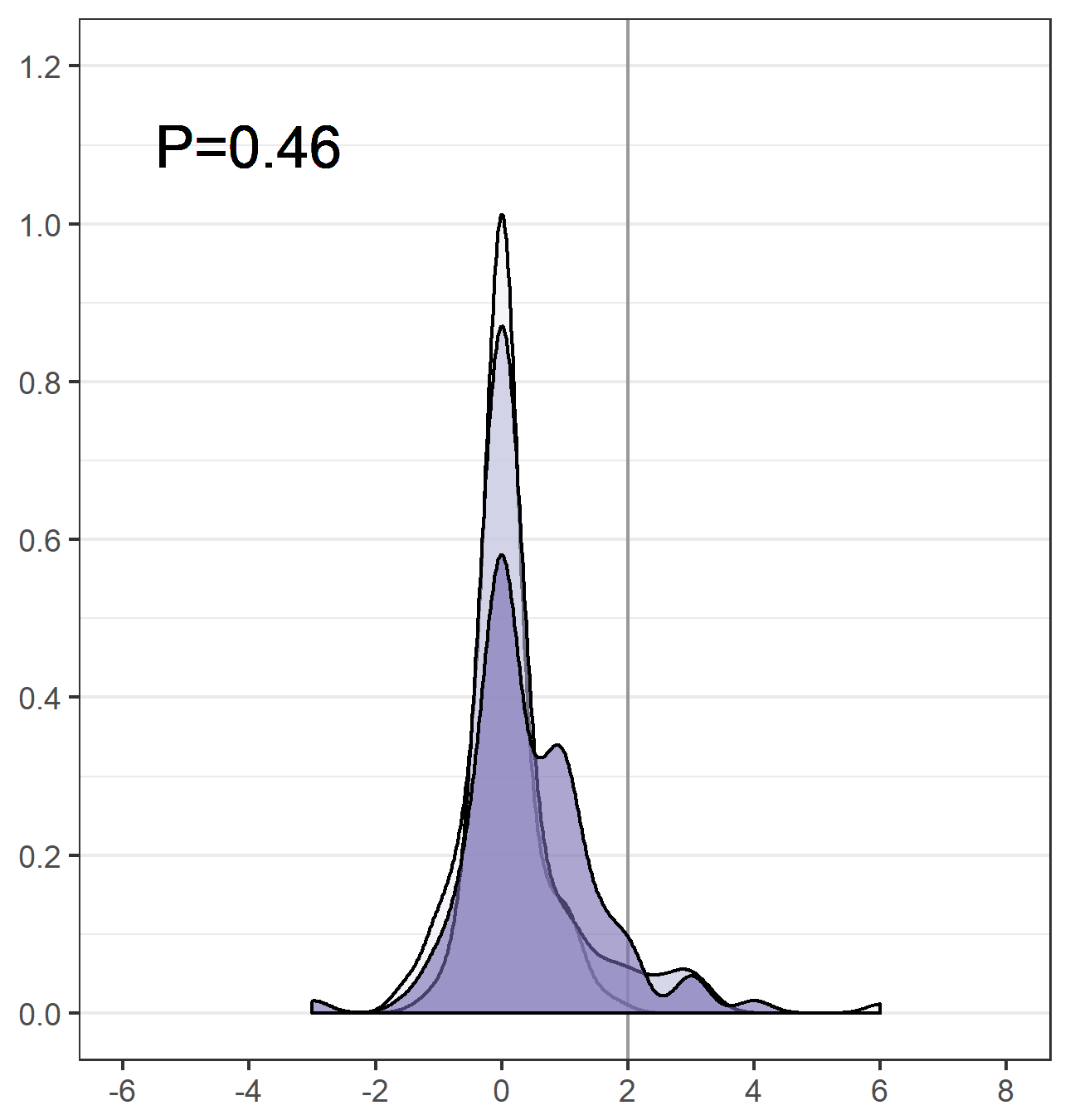 | 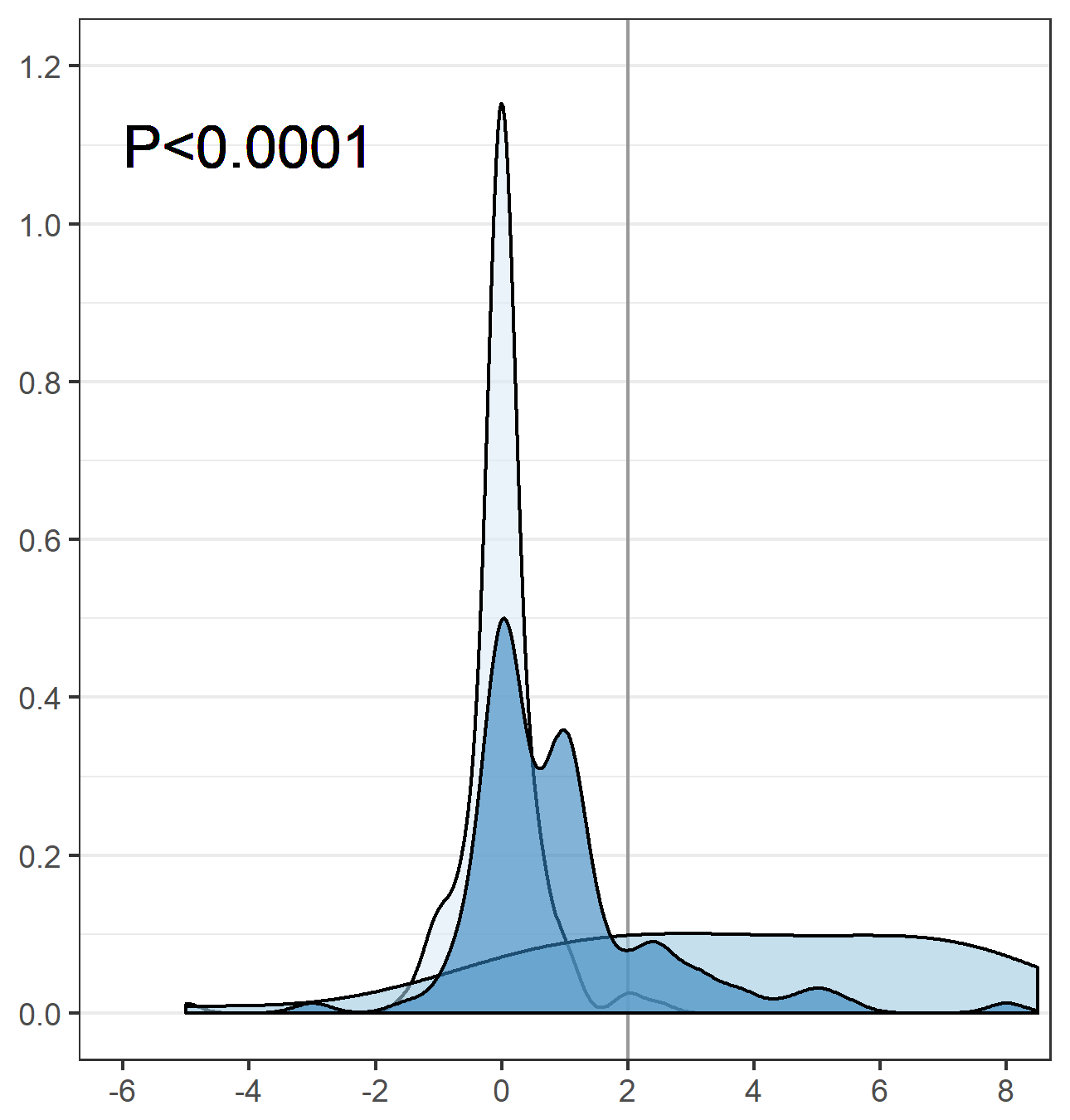 | 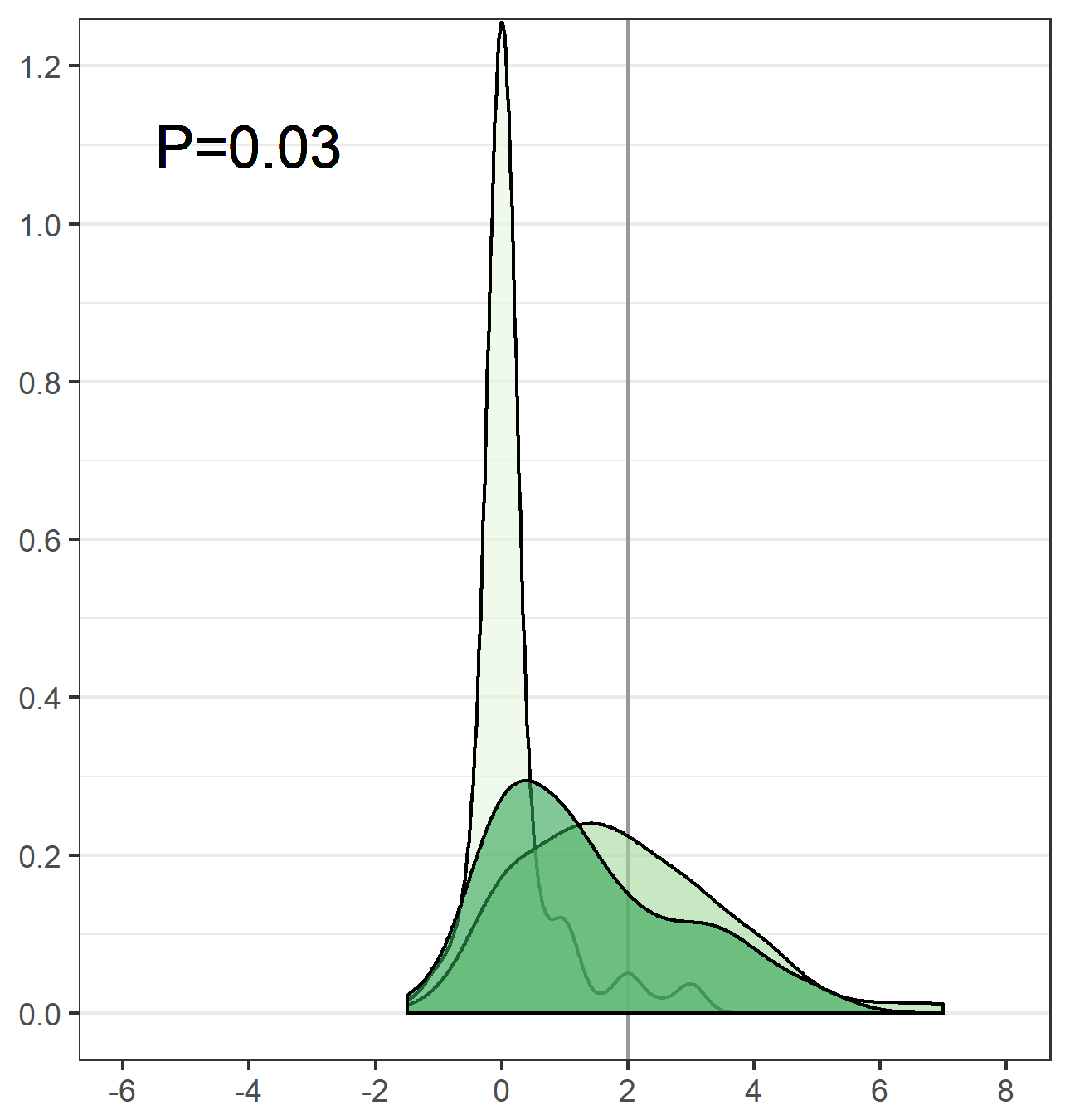 |
|  | **Serum Immunoglobulin G** | 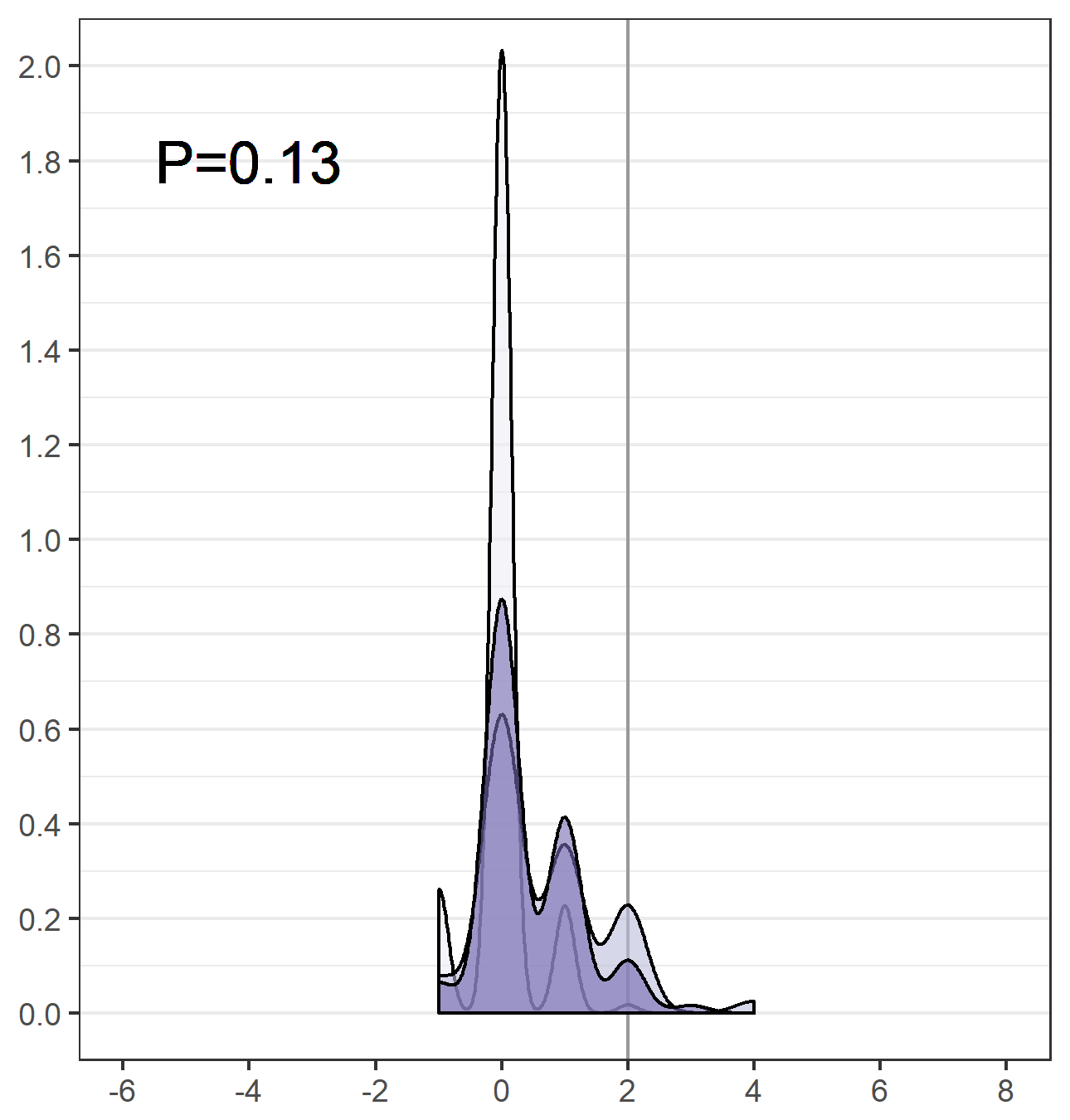 | 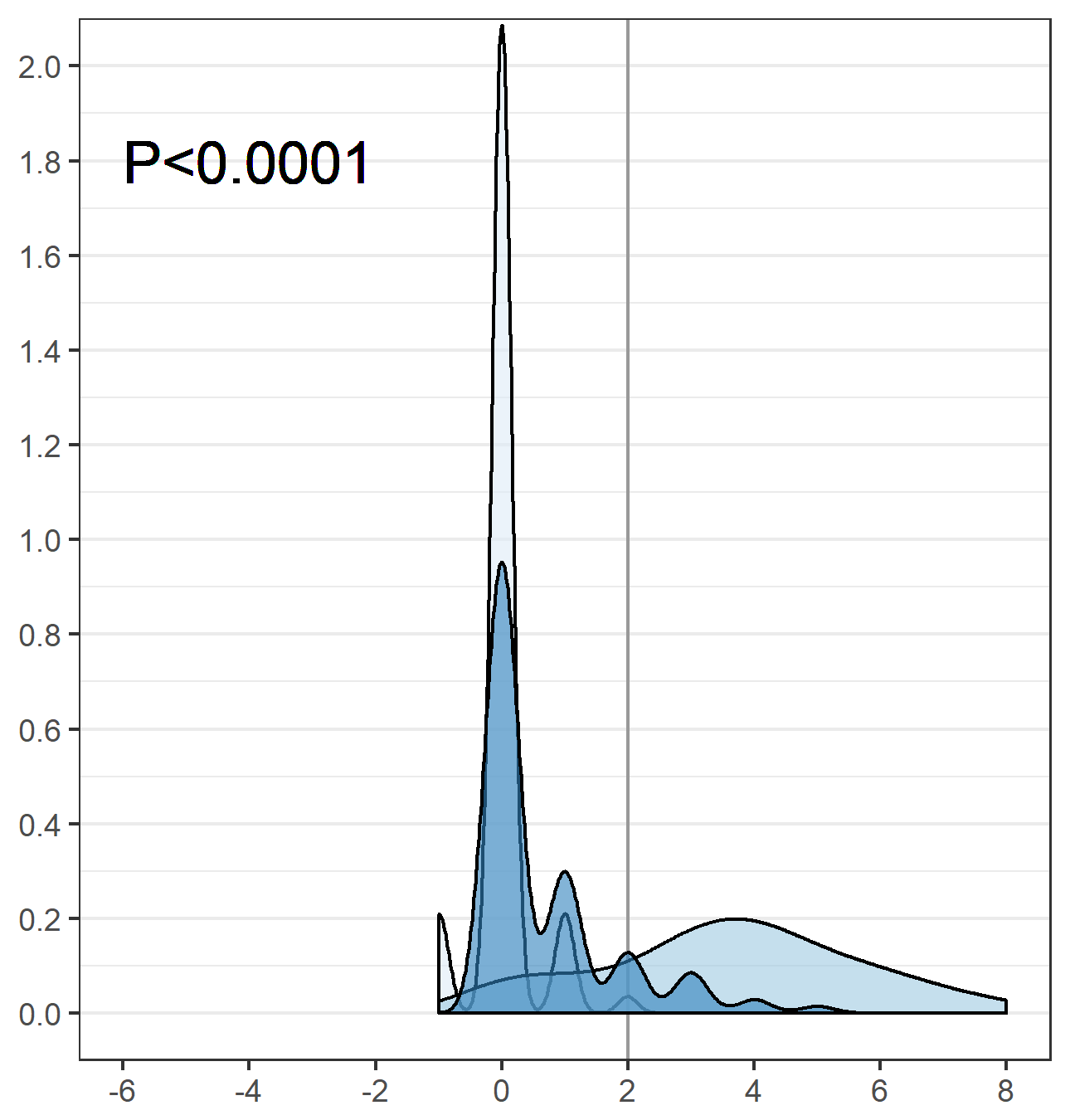 | 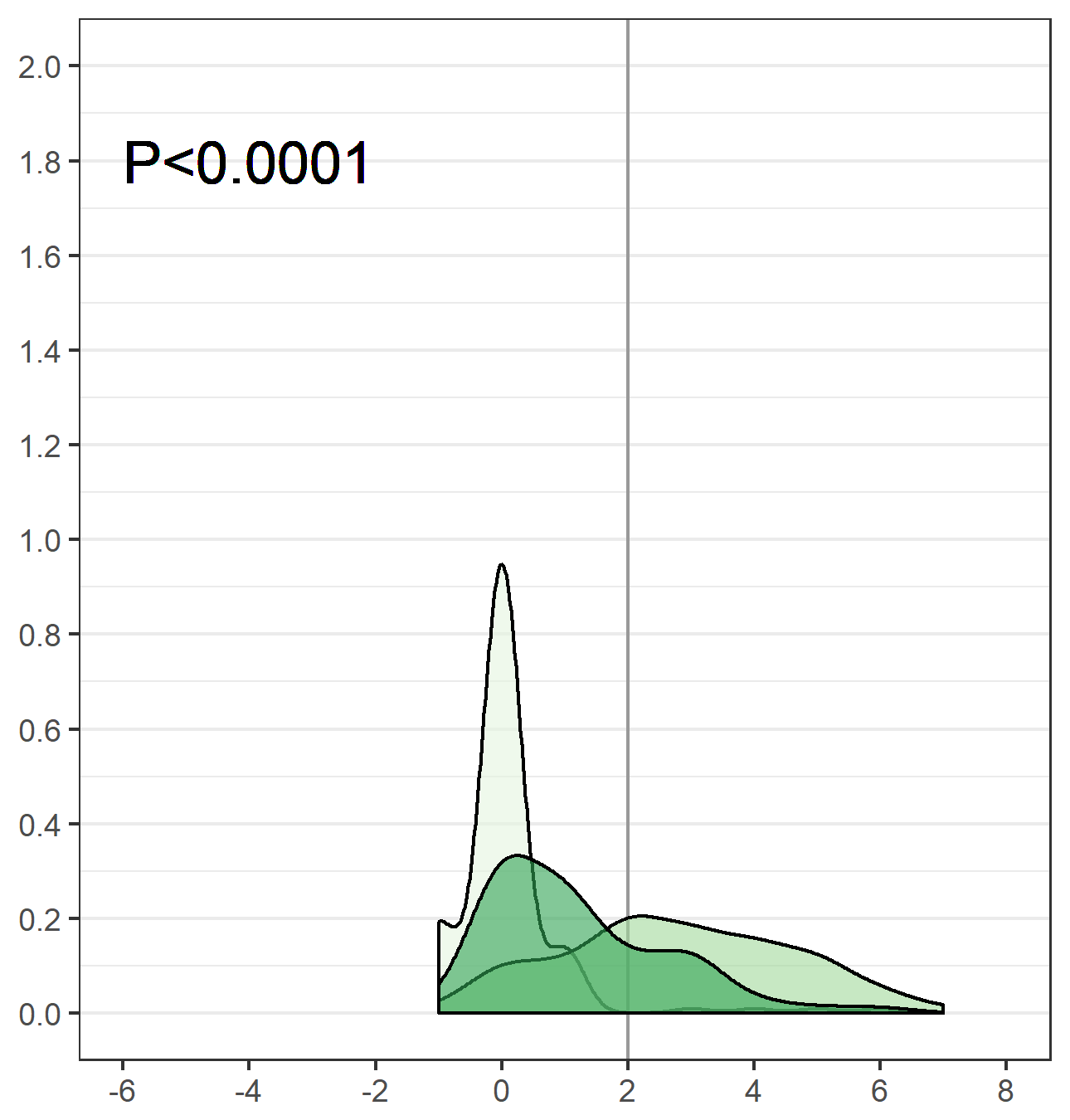 |
|  | **Serum Immunoglobulin A** | 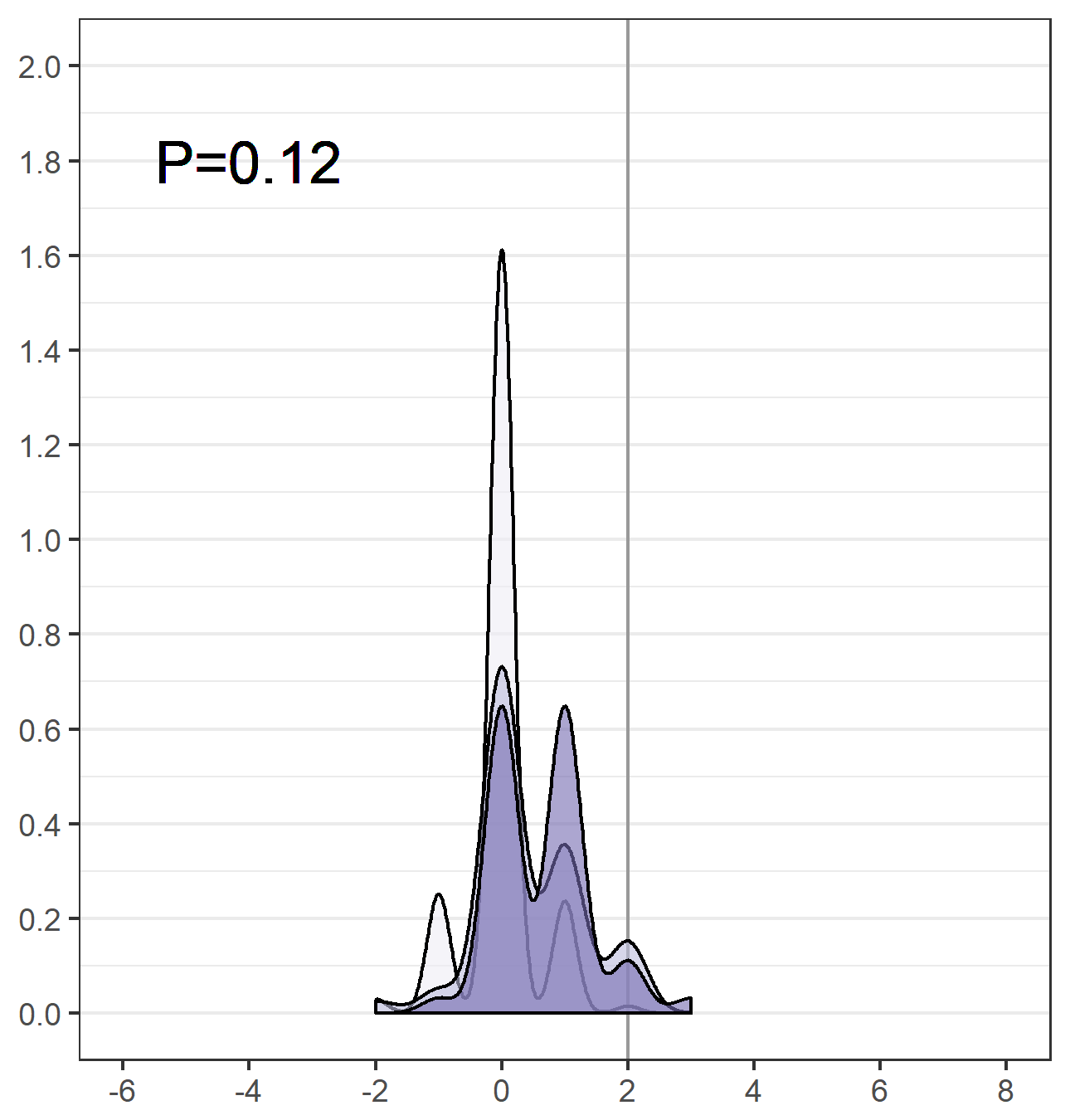 | 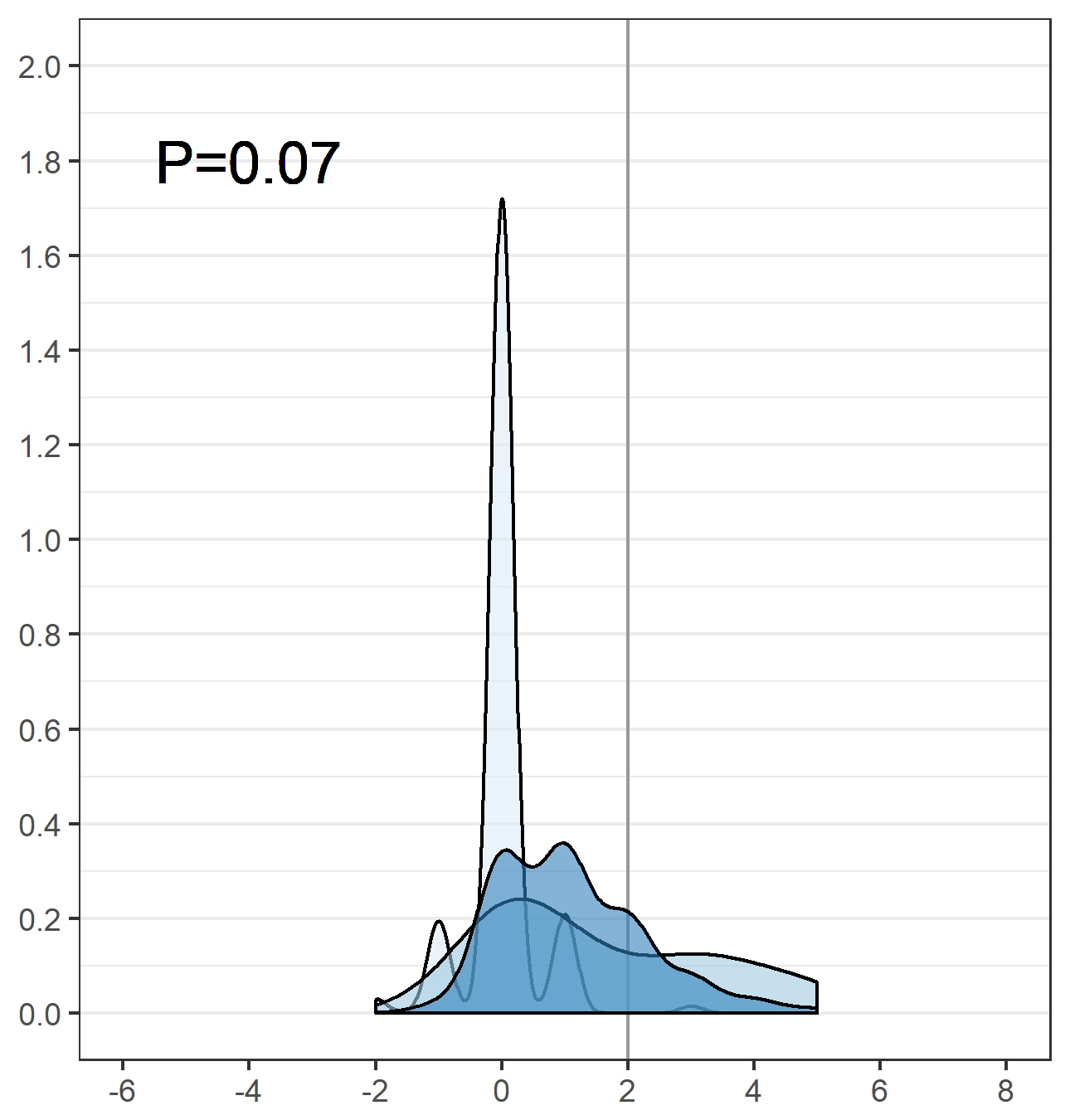 | 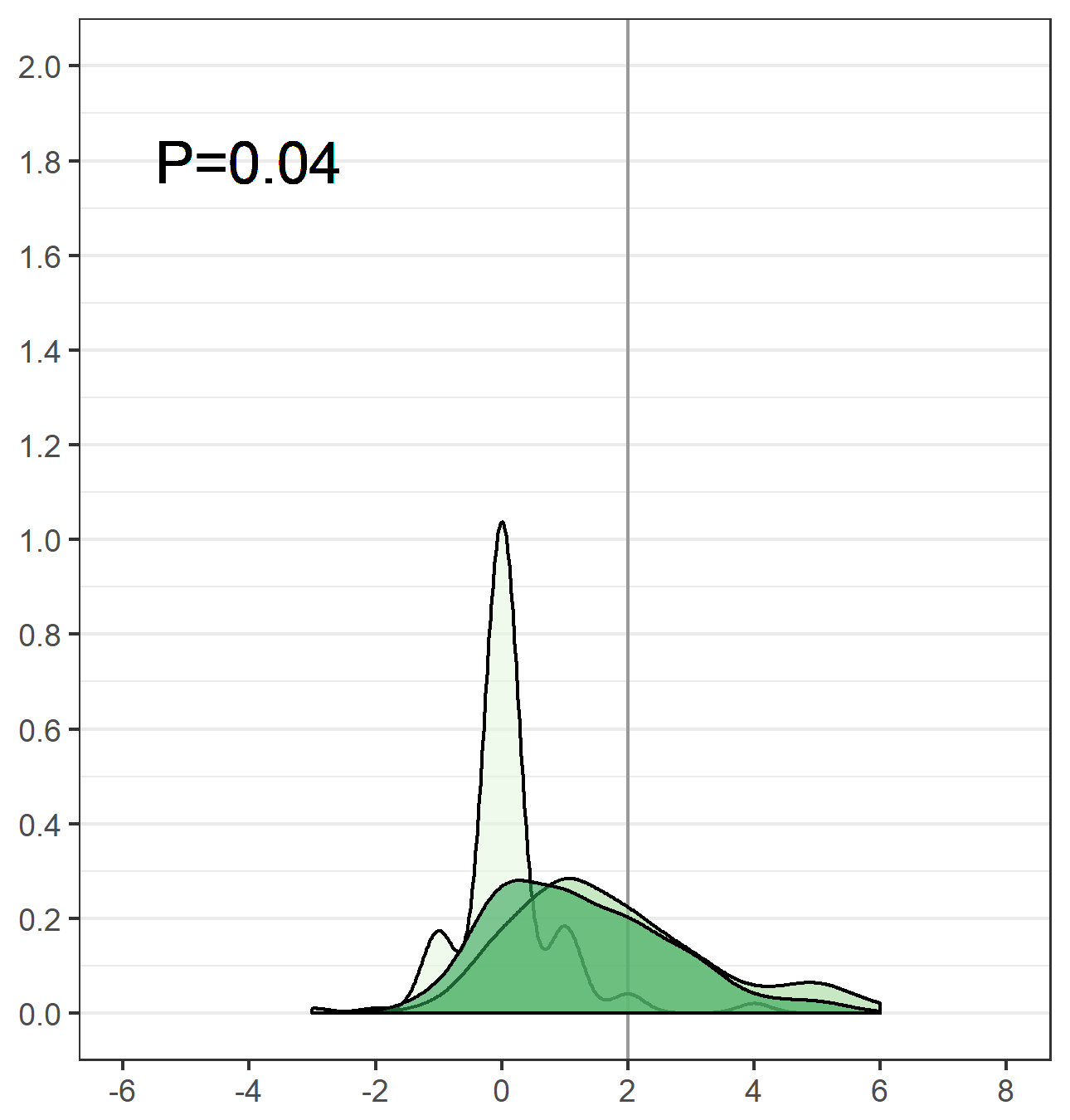 |
|  | **Mucosal Immunoglobulin A, Normalized** | 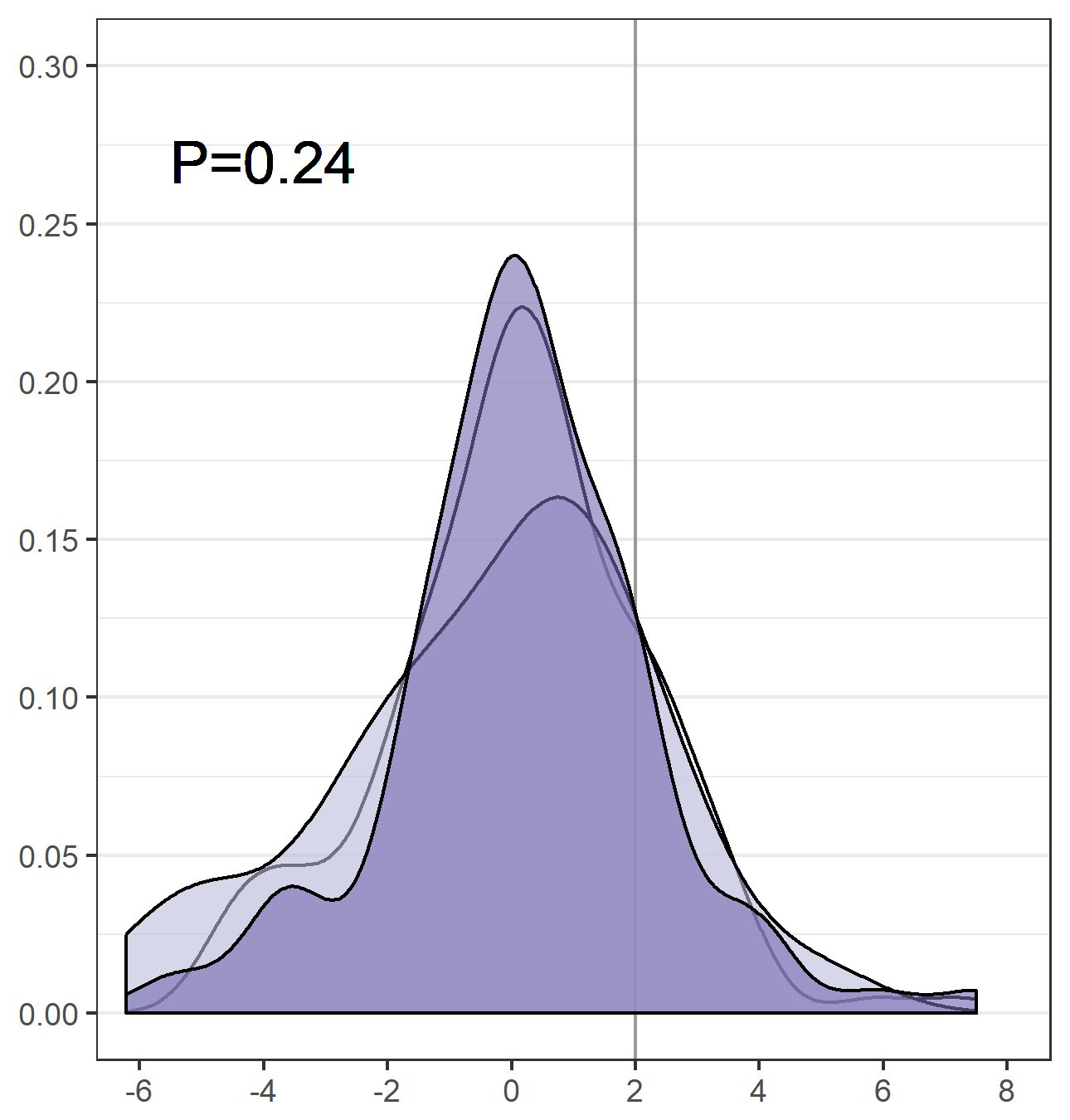 | 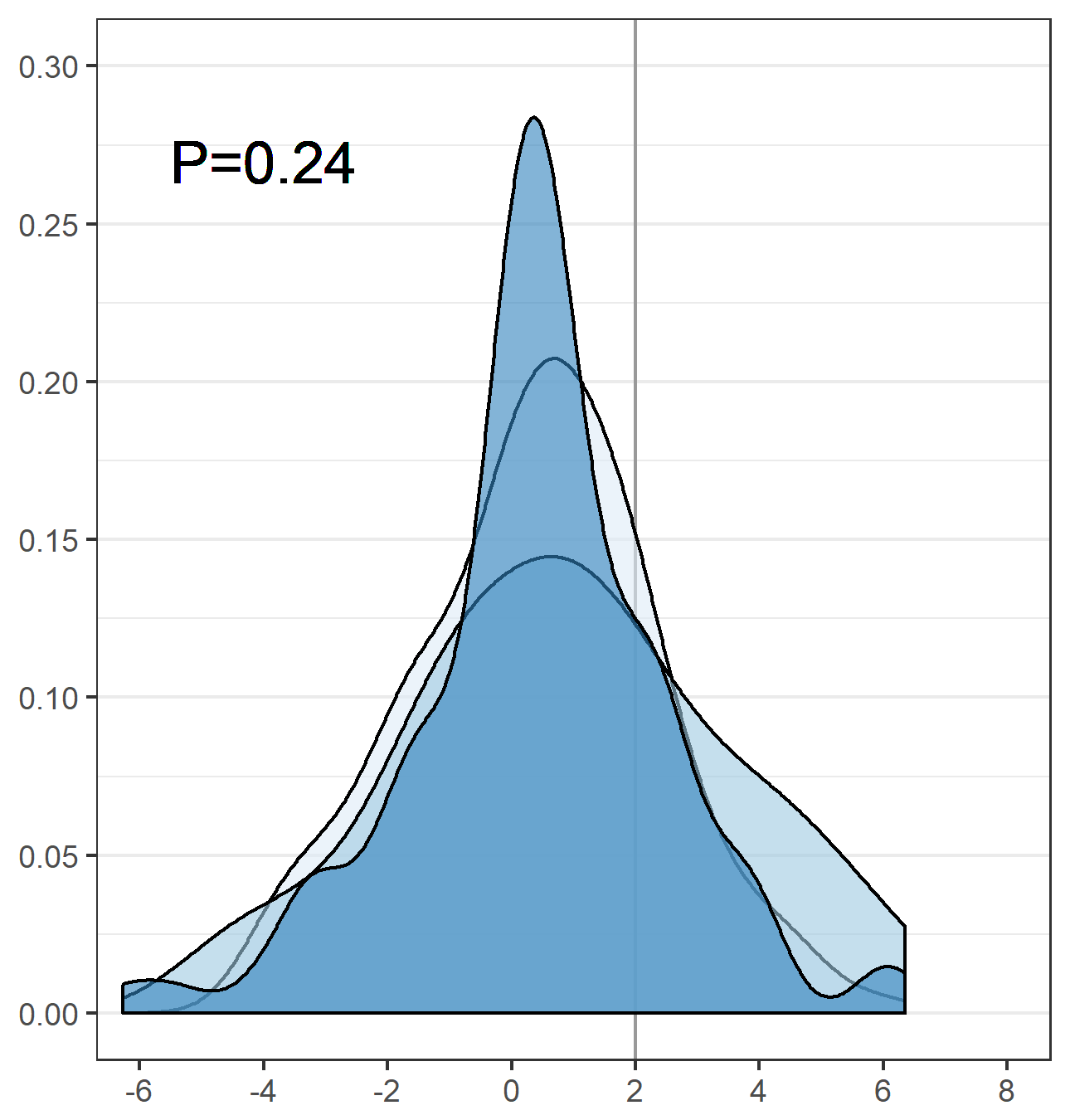 | 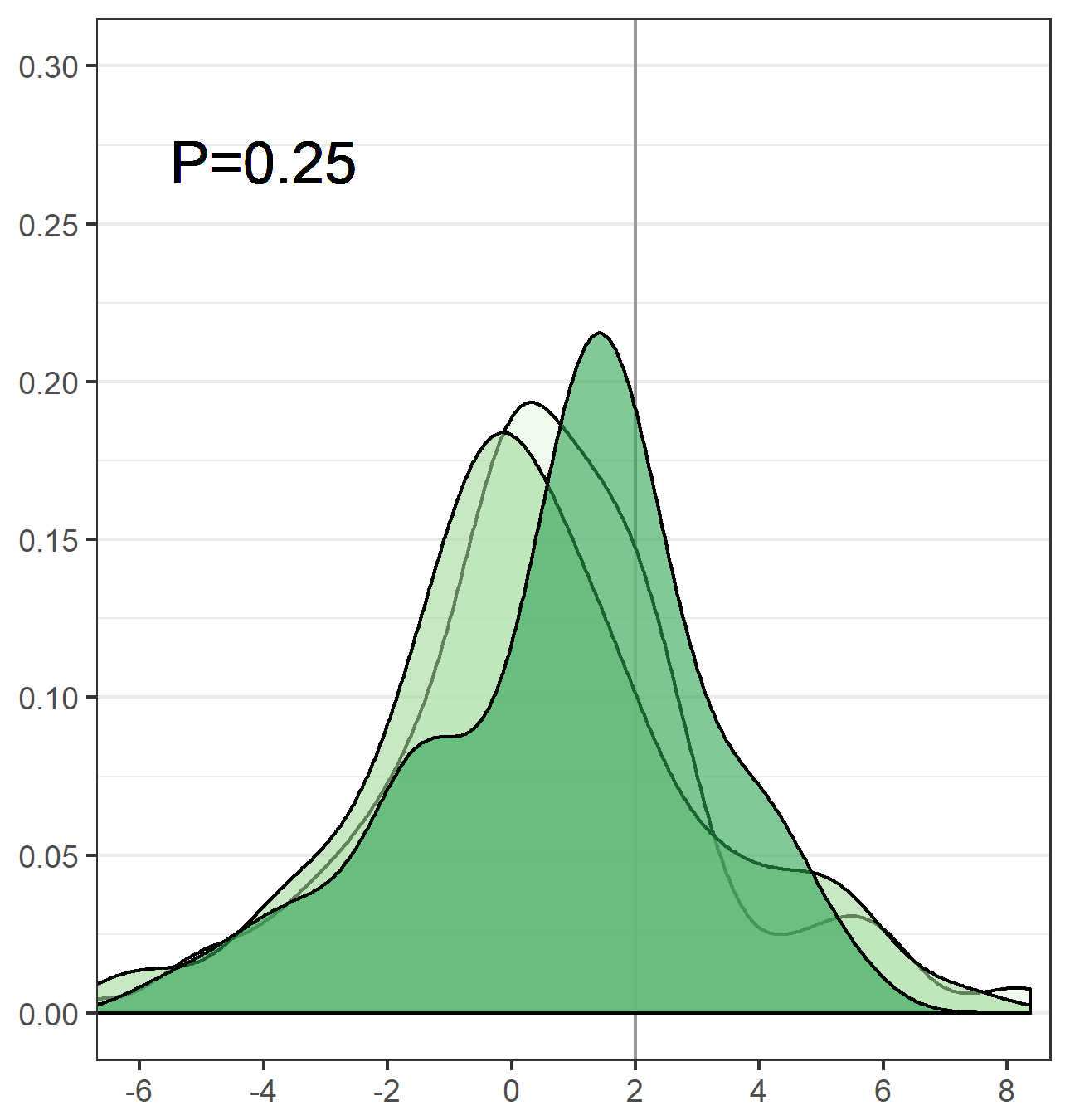 |
|  |  | **Difference in Log_2_ Titer between Days 21 and 0** | | |

**Supplementary Figure 2. Density plots illustrating the distributions of differences in the log_2_ serum and mucosal antibody titers between trial days 0 and 21 by immunoassay, influenza strain, and treatment/pre-existing HAI status at baseline (N=290).** P values are from likelihood ratio tests and indicate effect modification of the immune responses by baseline HAI status (i.e., HAI titer of <10 or ≥10 on day 0). The vertical reference lines indicate four-fold rises (i.e., a log_2_ difference ≥2) between the time points.

**Supplementary Table 1. Ranges of strain-specific serum and mucosal antibody titers in LAIV recipients by tertile at baseline (N=145).**

| Immunoassay | Strain | Bottom Tertile | Middle Tertile | Highest Tertile |
| --- | --- | --- | --- | --- |
| Serum HAI | A/H3N2 | 5 - 20 | 28 - 40 | 57 - 320 |
|  | B | 5 - 5 | 7 - 20 | 28 - 320 |
| Serum Microneutralization | A/H3N2 | 5 - 80 | 95 - 320 | 403 -2560 |
|  | B | 5 - 5 | 10 - 28 | 40 - 640 |
| Serum Immunoglobulin G | A/H3N2 | 50 - 3200 | 6400 - 12,800 | 12,801 - 25,600 |
|  | B | 100 - 800 | 1600 - 6400 | 12,800 - 25,600 |
| Serum Immunoglobulin A | A/H3N2 | 50 - 100 | 200 - 200 | 400 - 3200 |
|  | B | 50 - 100 | 200 - 200 | 400 - 1600 |
| Mucosal Immunoglobulin A, Normalized | A/H3N2 | 0.01 - 0.41 | 0.43 - 0.95 | 0.97 - 18.1 |
|  | B | 0.01 - 0.31 | 0.33 - 0.84 | 0.86 - 16.9 |
